# Supplementary material for: An essential Staphylococcus aureus cell division protein directly regulates FtsZ dynamics
Source: eLife. 2018 Oct 2;7:e38856. doi: 10.7554/eLife.38856 (PMC6168285; doi:10.7554/eLife.38856)
Supplement: Transparent reporting form [file elife-38856-transrepform.docx]

***eLife’s* transparent reporting form**

We encourage authors to provide detailed information *within their submission* to facilitate the interpretation and replication of experiments. Authors can upload supporting documentation to indicate the use of appropriate reporting guidelines for health-related research (see [EQUATOR Network](http://www.equator-network.org/%20)), life science research (see the [BioSharing Information Resource](https://biosharing.org/)), or the [ARRIVE guidelines](http://www.plosbiology.org/article/info:doi/10.1371/journal.pbio.1000412) for reporting work involving animal research. Where applicable, authors should refer to any relevant reporting standards documents in this form.

If you have any questions, please consult our Journal Policies and/or contact us: [editorial@elifesciences.org](mailto:editorial@elifesciences.org).

**Sample-size estimation**

- You should state whether an appropriate sample size was computed when the study was being designed
- You should state the statistical method of sample size computation and any required assumptions
- If no explicit power analysis was used, you should describe how you decided what sample (replicate) size (number) to use

Please outline where this information can be found within the submission (e.g., sections or figure legends), or explain why this information doesn’t apply to your submission:

Microscopy data.

No explicit power analysis was used, but computing data from 20-500 cells is typically reported in the field for microbial microscopy images.

Line 133: n=3 replicates for transformation efficiency experiment

Line 144, Fig. 2A-D: n=676 cell diameters measured in microscopy images

Line 144, Fig. 2A-D: n=100 cell diameters measured in microscopy images for negative control.

Line 145, Fig. 2A-D: n=971 cell diameters measured in microscopy images

Line 146, Fig. 2E-F: n=770 cell diameters measured in microscopy images

Figure 2K. This was a manual quantification of cell diameters of 200 cells from microscopy images. No explicit power analysis was used. Computing data from 100-200 cells is typically reported in the field for microbial microscopy images.

Line 253, Fig. 4A-B: n=200 cell diameters measured in microscopy images

Line 259, Fig. 4C-D: n=200 cells examined for ZapA-GFP localization

Line 263, 264 (Fig. 4E-F): n=200 cells examined for GpsB-GFP localization

Line 273 (Fig. 4G): n=200 cells examined for ZapA-GFP localization

Line 275 (Fig. 4H-I): n=100 cells examined for ZapA-GFP localization

Line 279 (Fig. 4J): n=200 cells examined for ZapA-GFP localization

Line 282 (Fig. 4K): n=200 cells examined for ZapA-GFP localization

Line 283 (Fig. 4K): n=75 cells in which ZapA-GFP fluorescence intensity was measured; error reported as SD.

**Replicates**

- You should report how often each experiment was performed
- You should include a definition of biological versus technical replication
- The data obtained should be provided and sufficient information should be provided to indicate the number of independent biological and/or technical replicates
- If you encountered any outliers, you should describe how these were handled
- Criteria for exclusion/inclusion of data should be clearly stated
- High-throughput sequence data should be uploaded before submission, with a private link for reviewers provided (these are available from both GEO and ArrayExpress)

Please outline where this information can be found within the submission (e.g., sections or figure legends), or explain why this information doesn’t apply to your submission:

Biochemistry experiments:

Fig. 5 legend, lines 859-862; error bars defined as SEM, n=3 replicates stated; the box-and-whisker plots shown includes the entire range of data obtained, including any “outliers”, so that the reader may appreciate how mean values were calculated.

Fig. 6 legend, lines 869 and 879. We stated that the representative data shown was from greater than or equal to 3 independent experiments. We did not exclude any data from the analysis.

**Statistical reporting**

- Statistical analysis methods should be described and justified
- Raw data should be presented in figures whenever informative to do so (typically when N per group is less than 10)
- For each experiment, you should identify the statistical tests used, exact values of N, definitions of center, methods of multiple test correction, and dispersion and precision measures (e.g., mean, median, SD, SEM, confidence intervals; and, for the major substantive results, a measure of effect size (e.g., Pearson's r, Cohen's d)
- Report exact p-values wherever possible alongside the summary statistics and 95% confidence intervals. These should be reported for all key questions and not only when the p-value is less than 0.05.

Please outline where this information can be found within the submission (e.g., sections or figure legends), or explain why this information doesn’t apply to your submission:

Fig. 5 legend, lines 859-862; error bars defined as SEM, n=3 replicates stated.

Fig. 5F: Box-and-whisker plots were used to show the entire range of data obtained.

(For large datasets, or papers with a very large number of statistical tests, you may upload a single table file with tests, Ns, etc., with reference to sections in the manuscript.)

**Group allocation**

- Indicate how samples were allocated into experimental groups (in the case of clinical studies, please specify allocation to treatment method); if randomization was used, please also state if restricted randomization was applied
- Indicate if masking was used during group allocation, data collection and/or data analysis

Please outline where this information can be found within the submission (e.g., sections or figure legends), or explain why this information doesn’t apply to your submission:

Not applicable for bacterial image analyses in which isogenic strains were used.

Not applicable for in vitro biochemistry analyses in which purified proteins were used and experiments were performed at least in triplicate.

**Additional data files (“source data”)**

- We encourage you to upload relevant additional data files, such as numerical data that are represented as a graph in a figure, or as a summary table
- Where provided, these should be in the most useful format, and they can be uploaded as “Source data” files linked to a main figure or table
- Include model definition files including the full list of parameters used
- Include code used for data analysis (e.g., R, MatLab)
- Avoid stating that data files are “available upon request”

Please indicate the figures or tables for which source data files have been provided:

All numerical data have been reported in the main text when describing the figures. No large data sets were used in this manuscript.
